# Developing a Theoretically Informed Strategy to Enhance Pharmacist-Led Deprescribing in Care Homes for Older People

**DOI:** 10.3390/pharmacy13050133

**Published:** 2025-09-16

**Authors:** Linda Birt, David Wright, David P. Alldred, Christine M. Bond, Richard Holland, Carmel Hughes, Sion Scott

**Affiliations:** 1 School of Healthcare, University of Leicester, Leicester LE1 7RH, UK; linda.birt@leicester.ac.uk (L.B.); d.j.wright@leicester.ac.uk (D.W.); 2 School of Healthcare, University of Leeds, Leeds LS2 9JT, UK; d.p.alldred@leeds.ac.uk; 3 Primary Care, University of Aberdeen, Aberdeen AB24 3FX, UK; 4 Exeter Medical School, University of Exeter, Exeter EX4 4PY, UK; 5 School of Pharmacy, Queen’s University of Belfast, Belfast BT7 1NN, UK

**Keywords:** medicines optimisation, medicine review, polypharmacy, overprescribing, long-term care home, older people, behaviour change

## Abstract

Polypharmacy is prevalent in older people residing in care homes. Deprescribing, reducing or stopping harmful or unnecessary medicines, leads to improvements in patient- and health-system-orientated outcomes. This study identified the barriers and enablers to pharmacists proactively deprescribing in United Kingdon care homes. It draws on methods from behavioural science. Twenty-nine participants who had previously taken part in a deprescribing randomised control trial (sixteen pharmacists, six primary care doctors, and seven care home managers) were interviewed. Data were mapped to the Theoretical Domains Framework to understand pharmacists’ deprescribing behaviour. Barriers were deprescribing seen as risky and perceived resistance to deprescribing by residents, their families, and care home staff. Enablers were seeing benefits from deprescribing, part of a pharmacists’ role, and endorsement from a doctor. Ways to change pharmacist behaviour were identified from a suite of behaviour change techniques (BCT). Using a modified Nominal Group Technique, 15 staff (six pharmacists, five primary care doctors, and four care home managers) naïve to deprescribing interventions completed an online survey to assess the feasibility and acceptability of implementing the 27 BCTs. Seven BCTs achieved a more that 80% consensus on all implementation criteria. In a consensus workshop, the staff group discussed practical ways the BCTs might work in primary care practice. Fourteen UK policy and practice leaders worked with the researchers to develop recommendations from the consensus workshop into a policy briefing. In conclusion, this study provides detail on using a theory-informed approach to translate research into policy to inform deprescribing practices.

## 1. Introduction

Polypharmacy is considered the prescribing of five or more medicines and there is robust evidence that older people in care homes are at great risk of polypharmacy [1,2,3]. Polypharmacy can lead to an increased risk of drug–drug interactions [2], adverse drug events such as falls [4,5], and an increased drug burden [6].

Proactively deprescribing, i.e., reducing or stopping inappropriate medicines or those no longer needed, is a complex behaviour and for the activity to be successful, barriers and enablers to the behaviour require addressing [7]. The healthcare professional, often a pharmacist, needs to know which medicines to deprescribe, be motivated to deprescribe, and have the opportunity to complete the activity [8]. There also needs to be a consensus across staff working in care homes and the wider primary care team that deprescribing is required and will lead to benefits [9]. There is a need to educate residents and families on the risks of polypharmacy and potential benefits of deprescribing so that they are receptive to deprescribing [10].

Several interventions designed to increase deprescribing behaviour have defined pharmacists as a healthcare professional group who are well-placed to deprescribe [11,12]. Some pharmacists may have additional qualifications to allow them to make deprescribing decisions autonomously, whilst others make deprescribing recommendations to another healthcare professional, e.g., a primary care doctor, who may then enact the deprescribing decision. For the latter scenario, there is increasing evidence that in many cases, deprescribing recommendations are not enacted [13]. This can be for a variety of non-clinical reasons such as recommendations being missed or insufficient time to enact them [13].

The Care Homes Independent Prescribing Pharmacist Study (CHIPPS) evaluated the effectiveness and cost-effectiveness of pharmacists assuming responsibility for medicines management in care homes for older people in the United Kingdom (UK) [14]. The CHIPPS found that Pharmacist Independent Prescribers (PIPs) could safely and effectively deprescribe medications for older people within UK care homes [15]. However, a secondary analysis of CHIPPS data identified that there were contextual factors which influenced the deprescribing activity [8]. Contextual factors associated with increased deprescribing activity were an established relationship between the pharmacist and care home, the endorsement of pharmacist deprescribing by the general practitioner(s) (GPs) responsible for the care home, and pharmacists assuming responsibility for all medicine management activities in the care home [8].

While there is international evidence, in the trial context, for the safety and effectiveness of pharmacist deprescribing for older people [16], there is less evidence examining the translation of trial interventions into everyday practice. Therefore, the ‘Learning from CHIPPS-moving to policy’ study aimed to re-interview CHIPPS participants, a year after the trial finished to examine ongoing PIP deprescribing in care homes for older people, followed by using behavioural science methods to develop strategies to address identified barriers and enhance enablers. Additionally, we aimed to include patient and public involvement and engagement (PPIE) and policy-maker stakeholders to develop policy guidance, designed to facilitate the adoption of the identified behaviour change strategies across care homes in the UK.

## 2. Materials and Methods

### 2.1. Study Context

This 12-month study ran from April 2021–March 2022. Ethical approval was granted by the University of East Anglia, Faculty of Medicine and Health Sciences Research Ethics Committee.

### 2.2. Patient and Public Involvement and Engagement

Two PPIE partners contributed to all stages of the research: one had lived experience of being prescribed polypharmacy and having medicines deprescribed and the other was the carer of such a person. They undertook, as a minimum, the following activities: (1) reviewed study documents and provided experiential comment on the analysis; (2) identified and validated themes within interview transcripts, focusing on enablers and barriers; (3) discussed the process of addressing barriers and enablers and reviewed the draft questionnaire for the Expert Panel; and (4) reviewed outputs and discussed dissemination activities.

Additionally, we convened a policy stakeholder group comprising of 14 policymakers, including representation from the Departments of Health in Northern Ireland, Scotland, and Wales; from NHS England and the Department of Health and Social Care; from Care England and the National Care Forum; and from the Parliamentary Office of Science and Technology. Additionally, there was representation from the Primary Care Pharmacy Association Care Homes Group, primary care pharmacy leads, Health and Social Care Boards, and PrescQuipp (which provides resources to support healthcare staff in medicine management). Prior to the policy stakeholder workshop, members were asked to review and consider critical comments on a draft policy briefing that the researchers had developed in collaboration with PPI colleagues. The first workshop was guided by a sequence of questions drawn from the National Institute for Health and Care Research (NIHR) policy writing guidance: [17]

1 What does and does not make a policy brief readable?
2 What should the ‘Policy Implications’ be from this brief?
3 How would policy makers implement it?
4 How do we disseminate this to the right people and have the recommendations implemented?

After the workshop, two more versions of the policy briefing were shared with the policy stakeholder group. There was good engagement and email suggestions on changes were considered by the research team with changes made as appropriate. The final document was then shared for wider dissemination.

### 2.3. Study Design

This study comprised of two phases: (1) interviews with participants who took part in CHIPPs to identify the enablers and barriers to deprescribing in a non-trial context and (2) a consensus panel of pharmacists, GPs, and care home (CH) managers who had not been involved in the CHIPPS study who undertook an online survey and workshop to develop a behavioural-science-underpinned strategy that addressed the barriers and enablers identified in Phase 1. Behavioural science examines why people behave in certain ways and what strategies might help them change the way they behave. For example, a pharmacist might undertake the behaviours of prescribing a medication because they have knowledge of how the medicine works, because they have authority to do this, and because they have previously seen the medicine help a patient. These three factors, knowledge, professional role, and belief about consequence, are the determinants that make prescribing behaviours happen. If these determinants are missing then a behaviour may not happen [18].

#### 2.3.1. Phase 1: Identification of Enablers and Barriers to Pharmacist-Led Deprescribing in Care Homes Using Interviews

Aim: To develop a theory-informed understanding of barriers and enablers to pharmacist-led deprescribing in care home

Recruitment: The samples were drawn from pharmacists, GPs, and CH managers who had taken part in the CHIPPS deprescribing intervention. They were sent an email invitation and participant information sheet. All who expressed an interest were interviewed.

Data collection: After signing an online consent form, the researcher arranged one-to-one virtual interviews using a pre-prepared topic guide underpinned by domains in the Theoretical Domains Framework (TDF) [19] (see Supplementary File S1). The TDF is a synthesis of 33 behavioural science theories; it aims to provide insights into appropriate behavioural change strategies [19].

Analysis: Data were initially analysed thematically using an inductive lens and barriers and enablers of the deprescribing identified; the methods were comprehensively reported elsewhere.]. Then, using a retroductive approach, data were mapped to the TDF containing 14 behavioural domains [19]. Using Excel™, small segments of interview data were cut and pasted under the relevant domain, thereby providing a visual record of the most relevant behavioural domains. To increase the dependability of the results, two researchers (LB & SS) mapped the data, reaching agreement on any difference. The trustworthiness of the results was further enhanced by peer validation with the wider research team. PPI representatives examined three interview transcripts and described the barriers and enablers that they could identify.

Following the mapping of the data to the TDF, we used the Theory and Techniques Tool (https://theoryandtechniquetool.humanbehaviourchange.org/ (accessed on 22 July 2021)), which links TDF domains to theory and evidence-based behaviour change techniques (BCTs), to identify all potential BCTs to address the identified barriers and enablers. A BCT is a strategy that helps an individual change their behaviour.

#### 2.3.2. Phase 2: Development of Behavioural-Science-Underpinned Strategy to Address the Barriers and Enablers Identified in Phase 1 Using a Consensus Panel

Aim: To facilitate PIPs and other stakeholders to select BCTs to include in a strategy to address the identified barriers and enablers.

Recruitment: We invited expressions of interest from PIPs, GPs, and CH managers naïve to the CHIPPS intervention to form a consensus panel. Invitations were shared through gatekeepers at a range of professional UK networks [e.g., the Primary Care Pharmacy Association Care Homes Group, Society for Academic Primary Care, and the Contact, Help, Advice, and Information Network (CHAIN)]. Recruitment was open to stakeholders in the three countries served by CHIPPS: England, Northern Ireland, and Scotland. We purposively sampled consensus panel members from the expressions of interest to facilitate a mix of professions and geographical diversity, including representation from urban, suburban, and rural contexts and representation from three UK nations.

Data collection: We used a modified Nominal Group Technique (NGT) approach to facilitate the selection and characterisation of BCTs from Phase 1. An NGT is a consensus method which can generate solutions to research questions through idea generation, problem solving, prioritisation, and agreement [20]. We used a form of modified NGT which drew on elements from Delphi methods, where geographically dispersed participants completed an online consensus survey (Stage 1) to inform an online NGT workshop (Stage 2), rather than several rounds of group deliberation [21].

##### Stage 1: Online Consensus Survey

Data collection:We produced plain English descriptions for each BCT identified in the Phase. In the survey, participants were asked to rate the BCT against the APEASE criteria: a checklist used to assess if a BCT strategy was feasible and appropriate [22]. We omitted affordability (A) as participants might not have been aware of costs.

The criteria used were as follows:

1. Whether it was **P**ractical to put in place in care homes.

2. The likelihood of being **E**ffective.

3. The **A**cceptability to everyone involved, e.g., other staff, residents, and family.

4. Whether it was likely to be **S**afe and free of undesirable consequences.

5. Whether it was **E**quitable, so not likely to increase disparities between residents or across settings.

The survey was tested with pharmacist and GP collaborators and refinements were made. PPI colleagues also reviewed the survey (see Supplementary File S2 for the survey). Following consent, participants were emailed a link to the Microsoft^®^ Forms platform to complete the online consensus survey. The survey estimated completion time was up to 1 h.

Data analysis:: In the survey, we set a consensus threshold of 80% of panel members agreeing that a BCT met all the -PEASE criteria [22]. All BCTs were categorised into one of the following groups:

(a) Accepted: BCTs where all five -PEASE criteria reached ≥80% agreement.
(b) Rejected: BCTs where one or more -PEASE criteria reached ≥80% disagreement.
(c) Requires consensus discussion: BCTs where some or all -PEASE criteria failed to reach ≥80% agreement and did not meet the threshold for rejection above.

BCTs categorised as ‘requires consensus discussion’ and ‘accepted’ would proceed to the Stage 2 online NGT for further discussion. BCTs categorised as rejected would be excluded from further consideration.

##### Stage 2: Nominal Group Technique Workshop

Data collection: The objectives of Stage 2 were to facilitate discussion to achieve a consensus to accept or reject any ‘requires consensus discussion’ BCTs from Stage 1. Then, to facilitate a discussion on how to characterise, in practical ways, the accepted BCTs for the deprescribing strategy. The participants who completed the Stage 1 online survey attended the NGT workshop.

## 3. Results

### 3.1. Phase 1: Identification of Enablers and Barriers to Pharmacist-Led Deprescribing in Care Homes Using Interviews

We undertook interviews with 29 participants who had been involved in the CHIPPS intervention (sixteen pharmacists, six GPs, and seven care home managers). See Table 1 for the sample characteristics. The findings of the thematic analysis are comprehensively reported elsewhere [23] and a summary is provided below.

The thematic analysis identified structural and individual barriers and enablers to deprescribing. The barriers included a lack of dedicated time within the PIP role, concern that deprescribing would have adverse effects on the residents’ wellbeing and behaviour. In contrast, the enablers were when deprescribing was seen as a key part of a clinical pharmacist’s role, seeing a benefit to a resident’s quality of life, when the pharmacist received support from GPs and primary care colleagues to review and stop medicines, and electronic prompts or reminders to review medicines.

Thematic analysis data were mapped to 12 domains of the TDF with most data aligning with the domains of social and professional roles and identity, social influence, an environmental context and resources, reinforcement, and beliefs about consequences. No data were mapped to the domains of behavioural regulation or intentions. Table 2 provides examples of data mapped to the main domains. Supplementary File S3 provides an example of the mapping process and illustrative quotes from the interview data. Following the mapping process, the research team identified three main barriers and three main enablers to pharmacist-led deprescribing, as shown in Table 2.

Using the Theory and Techniques Tool (https://theoryandtechniquetool.humanbehaviourchange.org/ (accessed on 22 July 2021)), we identified 27 BCTs linked to the TDF domains in Table 2 that could potentially be used to address the three barriers and three enablers. These 27 BCTs were written in plain English statements for the online survey used in Phase 2 (Supplementary File S2).

### 3.2. Phase 2: Development of Behavioural-Science-Underpinned Strategy to Address the Barriers and Enablers Identified in Phase 1 Using a Consensus Panel

We received 43 expressions of interest to join the consensus panel. Purposive sampling and the consideration of availability for attendance at the online consensus meeting led to a consensus panel of 15 people (six pharmacists, five GPs, and four CH managers); they represented locations across England and Scotland. All 15 consensus panel members completed the online consensus survey and 12 attended the workshop.

#### 3.2.1. Stage 1: Online Consensus Survey

Survey responses were analysed using the preset criteria of a consensus threshold of ≥80% agreement that a BCT met all five -PEASE criteria. Seven BCTs met the ≥80% agreement, with at least one for each barrier and enabler; Table 3 displays the seven BCTs which achieved over 80% consensus against all 5 -PEASE criteria.

As there was no need for silent deliberation in the workshop, the research team prepared slides to foreground the discussion on how these might be operationalised.

#### 3.2.2. Stage 2: Consensus Workshop

Twelve of the participants from stage 1 attended a 3 h online workshop (five pharmacists, four GPs, and three care home managers). After setting the scene, participants were distributed into small mixed professional groups each facilitated by a researcher with experience of behavioural change theory. Each group was asked to consider how a behaviour change strategy might be operationalised in practice. The activity was guided by three questions: What could the BCT strategy look like when implemented (content)? How might the strategy be delivered? How often and/or for how long? Ideas were noted in real time (see Supplementary File S4 for an example). After the workshop, the research team discussed the data for similarities and differences across the strategies. This led to a suite of recommendations on ways in which BCT strategies could be translated into practice (see Table 4). This new knowledge was developed into a policy briefing paper.

We sent the draft policy brief to the 14 members of the policy stakeholder group and convened a two-hour online meeting; those who could not attend the meeting were offered a one-to-one discussion. The meeting was a guided discussion [17]. The panel agreed on the key messages on behaviour change strategies and ways of implementing, but made several suggestions related to the language and layout. The research team acted on comments and after two further email consultations, the final policy briefing was produced. This is provided in Supplementary File S5 and is free for people to use. The panel supported the dissemination of the policy briefing.

## 4. Discussion

This study adds to the evidence on embedded barriers and enablers to pharmacist-led deprescribing in care homes. It makes important contributions to policy and potentially practice by providing theory-informed and stakeholder-developed behaviour change strategies to address deprescribing concerns and maximise positive behaviours and beliefs. We discuss the relevance of our results to practice at the level of the resident, clinician, and care system.

Although pharmacists have the knowledge to deprescribe, they may lack confidence, especially when families and residents disagree that a medicine has more risks than benefits [24,25]. Having time to develop relationships with residents may be important for pharmacists if they are to be able to adopt the behavioural change strategy of engaging with families and residents [8]. While improving a public understanding of the potential benefits of deprescribing is important, we found that there also needs to be positive reinforcement to the pharmacist in which the benefits outweigh the risks and that they have endorsement from other clinicians to undertake the activity. Education may be implemented through positive case studies which can be shared with residents and care home staff. Positive endorsement could be at the macro level, with recognition from medical and pharmacy professional bodies that deprescribing is a core pharmacist competency, and at the micro level, with feedback on positive consequences from peers, mentors, and primary care doctors [26].

In many situations, there is only one clinical pharmacist in the primary care team supporting the care home, which means peer support can be limited. This can create barriers to deprescribing because peer support may be useful in enhancing skills [26,27]. Training on deprescribing specific to older people and support from a mentor are key factors in increasing the pharmacist’s confidence and competency [28]. Where national care home pharmacist networks exist [29,30], these can be used to provide mentors, networking opportunities, and deprescribing toolkits. Alignment with similar care home bodies may also enhance their effectiveness.

Organisational structures and professional boundaries in care systems mean that doctors, pharmacists, and care home staff can have differing attitudes about deprescribing [31], which may mean pharmacist deprescribing decisions are not enacted [27]. Different professional knowledge of and attitudes to deprescribing may create ‘stop points’ in the deprescribing activity when doctors disagree with pharmacist decisions [32]. For optimum deprescribing in care homes, there needs to be open communication between the pharmacist, the primary care team, and the care home so clinical decisions can be shared, checked, and monitored for their effect on the care home resident. This aligns with the behavioural change strategy of ensuring there is a whole-team approach so pharmacists are supported. If clinical pharmacists are integrated into primary care teams, this can support professional trust and communication and improve medication management [32].

The ‘Learning from CHIPPS-moving into policy’ study aimed to bridge the gap between everyday deprescribing practice and research interventions which report positive outcomes from pharmacist-led deprescribing interventions in care homes. Working with professionals involved in and impacted by deprescribing and using a theory-informed method, we consider, should increase the likelihood of policy recommendations impacting on pharmacist-led deprescribing. Nonetheless, there are individual and organisational factors which might block the implementation into practice. On an individual level, deprescribing for older people requires specialised knowledge of how medicines affect older people, so even with endorsement and advise on how to involve families and residents in the activity, pharmacists may feel unskilled. Organisational challenges lie with financial resources and clear lines of endorsement; these often change with new funding contracts and systems of providing primary care. The ideas for implementation cluster around feedback on their performance, the opportunity for education, and endorsement from opinion leaders. These strategies align with implementation strategies found to be effective in a review exploring the effect of strategies designed to promote professional behaviour changes in healthcare staff [33].

### Limitations

These results need to be considered within the limitations of this study. The samples were drawn from health and social care staff who had an interest in the topic, so while they identified barriers, pharmacists who have not deprescribed in care homes may experience more or different barriers. There was little representation from care home staff or residents and their families, groups who often have less knowledge about the reasons for deprescribing. Future work should seek the views of residents and their families either in public involvement or, preferably, as participants in order to understand what drives their experiences and choice about stopping a medicine.

## 5. Conclusions

Our results add to the growing knowledge on how practitioners might address iatrogenic harm from prescribed medication through deprescribing, which is a priority for policy makers globally. For the UK context, this programme of research has directly responded to this priority by formulating a deprescribing strategy for pharmacist-led deprescribing in care homes. The use of behavioural change science provides a theoretical base for policy recommendations. The World Health Organisation emphasises that the success of any initiatives to reduce harm related to medications will depend on the extent to which it is a priority within health care systems. By aligning this programme of research with specific priorities set by the administrations across all four UK nations, and working closely with primary care practitioners alongside national policy leaders, we have taken steps towards bridging the gap between trial intervention and everyday practice. These evidence-informed strategies provide a practical roadmap for embedding pharmacist-led deprescribing into everyday care home practice.

## 6. Patents

There are no patents and the policy document can be freely used.

## Figures and Tables

**Table 1:** Interview sample characteristics.

Professional Group	Location	Length of Time Qualified as Independent Prescriber	Type of General Practice (GP)	Type of Care Home
Pharmacist Independent Prescribers n= 16	Scotland n = 5 N Ireland n = 4 England n = 7	≤5 years n = 8 6–10 years n = 6 ≥11 years = 2	-	-
General Practitioners n = 6	3 Scotland 1 N Ireland 2 England	-	Rural n = 2 Urban n = 4	-
Care home managers n = 7	3 Scotland 2 N Ireland 2 England	-	-	Residential n = 2 With nursing n = 5

**Table 2:** Barriers and enablers from interview data linked to the Theoretical Domains Framework.

Barrier or Enabler Statement	Illustrative Quote from Interview Data	Theoretical Domains Framework—Domain
1 Barrier: perceived resistance from residents and/or family members	*‘Or the most challenging thing is the relatives … if you take something away sometimes it can be seen that you’re not doing the best for their relative and that’s not an idea that they really like’ (PIP6)*	Social influence
2 Barrier: deprescribing is risky	‘*Downside is you are pushing for the sake of saying that you have deprescribed and I am quite against that, you know if we are not careful and that is what I fear might come with some medication reviews* etc. *if it becomes a tick box exercise with what you have reduced’* (PIP1)	Beliefs about consequences
3 Barrier: perceived resistance from care home staff regarding deprescribing some medicines	[CH staff reaction to reducing antipsychotics] *‘a little bit of resistance there like, “Well, you’re not going to touch that, are you?” Or, “Please leave that well alone.”* (PIP9)	Social influence
1 Enabler: observing the positive effects of deprescribing	*‘There was a patient who was falling, the anticholinergic burden score was really high, I managed to really reduce that and it felt like you were doing something really beneficial it was what the patient wanted, it was what the relatives wanted, yeah it’s a nice feeling to know that you are hopefully preventing falls’* (PIP4)	Reinforcement
2 Enabler: deprescribing is part of a pharmacist’s role	‘*Utilizing your skills and knowledge and where you’re at from a medication review point of view, by far I would say pharmacists are best-placed for that’* (PIP10)	Professional role/identity
3 Enabler: recognition and endorsement from the general practitioner that deprescribing is a pharmacist role	*‘Deprescribing. It is something that I look to be doing and that I am encouraged to do by my GP colleagues as well’ (PIP1)*	Social influence

**Table 3:** From online survey, the seven behaviour change techniques which achieved 80% consensus on all 5 -PEASE criteria.

Behaviour Change Techniques in Plain English	APEASE Criteria	
Barrier 1: Pharmacists are worried that residents, and/or their families may not want to stop medication. Strategy 1: A way of showing the pharmacist other pharmacists who have had discussions with residents and relatives in order to successfully deprescribe.	Practical	97.1
	Effective	83.3
	Acceptable	100
	Safe	91.7
	Equitable	91.7
Barrier 2: Pharmacists think that deprescribing is generally riskier than continuing to prescribe a medication, even if there are no anticipated future gains. Strategy 2: Emphasise the benefits of deprescribing and harmful consequences of failing to deprescribe in terms which will resonate with pharmacists.	Practical	91.7
	Effective	91.7
	Acceptable	100
	Safe	100
	Equitable	100
Barrier 3: Pharmacists are worried that some care home staff may be resistant to deprescribing. Strategy 2: Provide evidence to the pharmacist that the vast majority of care home staff are supportive of deprescribing.	Practical	100
	Effective	100
	Acceptable	100
	Safe	91.7
	Equitable	100
Barrier 3: Pharmacists are worried that some care home staff may be resistant to deprescribing. Strategy 4: Arrange for pharmacists to receive practical help from a colleague to work with care home staff to deprescribe.	Practical	83.3
	Effective	91.7
	Acceptable	100
	Safe	91.7
	Equitable	100
Enabler 1: Pharmacists believe that deprescribing for residents will lead to benefits. Strategy 2: Arrange praise for pharmacists whose deprescribing positively impacts a resident’s health and or wellbeing.	Practical	91.7
	Effective	91.7
	Acceptable	91.7
	Safe	83.3
	Equitable	91.7
Enabler 2: Pharmacists see deprescribing as a key part of their professional role. Strategy 1: Arrange for pharmacists to receive encouragement to deprescribe.	Practical	83.3
	Effective	91.7
	Acceptable	100
	Safe	100
	Equitable	100
Enabler 3: Endorsement by the general practitioner supports pharmacist deprescribing. Strategy 4: A way of showing pharmacists that general practitioners approve of deprescribing being a part of their role.	Practical	91.7
	Effective	91.7
	Acceptable	100
	Safe	100
	Equitable	100

**Table 4:** Characterisation of behaviour change techniques and how they may be operationalised.

Barriers and Enablers to Pharmacist-Led Deprescribing in Care Homes from Interview Data	Behaviour Change Strategy from Online Survey	Consensus Workshop Ideas for Implementation into Practice
Pharmacists are worried that residents and/or their families may not want to stop medication (barrier)	A way of showing the pharmacist other pharmacists who have had successful discussions with residents and their families in order to effectively deprescribe	Use carefully crafted films, including care home staff, pharmacists, and residents engaging in deprescribing consultation Mentoring by an experienced pharmacist or GP support from colleague (shadow)
Pharmacists think that deprescribing is generally riskier than continuing to prescribe even if there are no anticipated future gains (barrier)	Emphasise the benefits of deprescribing and the harmful consequences of failing to deprescribe in terms which resonate with the pharmacist	Involve local or national deprescribing networks Local could be multidisciplinary network, for example, care home medicine optimisation network where deprescribing experiences are shared
Pharmacists are worried that some care staff may be resistant to deprescribing (barrier)	1. Provide evidence to the pharmacist that the majority of care home staff are supportive of deprescribing 2. Arrange for the pharmacist to receive practical help from a colleague to work with care home staff to deprescribe	Develop care home medicine optimisation network to discuss professionals’ views and experiences Care home staff positive feedback
Pharmacists believe that deprescribing for residents will lead to benefits (enabler)	Arrange for feedback and recognition for pharmacists whose deprescribing positively impacts residents’ health and/or wellbeing	Build reflective links into professional practice with network Mentor to give feedback on good-quality deprescribing Care home medicine optimisation network that could be hosted nationally and/or locally
Pharmacists see deprescribing as a key part of their role (enabler)	Arrange for pharmacists to receive encouragement to deprescribe	Mentor to give feedback on good-quality deprescribing
Endorsement by GP supports pharmacist deprescribing (enabler)	A way of showing pharmacists that GPs approve of deprescribing being part of their role	Engage professional bodies, e.g., Royal College of General Practitioners, to formally endorse that deprescribing is a part of the pharmacist’s role Deprescribing to be formally incorporated into pharmacist’s job description

## Data Availability

The datasets generated and analysed during this study, as well as the protocols, are available from the corresponding author upon reasonable request.

## Supplementary Materials

The following supporting information can be downloaded at: https://www.mdpi.com/article/10.3390/pharmacy13050133/s1, Supplementary File S1: Topic guides; Supplementary File S2: Online survey; Supplementary File S3: Example of data mapping; Supplementary File S4: Example of consensus work shop material; Supplementary File S5: Policy briefing.

## Abbreviations

The following abbreviations are used in this manuscript:

BCT	Behaviour Change Technique
CHIPPS	The Care Homes Independent Pharmacist Prescribing Study
GP	General Practitioner
NGT	Nominal Group Technique
NIHR	National Institute for Health and Care Research
PIP	Pharmacist Independent Prescriber
TDF	Theoretical Domains Framework
UK	United Kingdom
