# Supplementary material for: Developing a Theoretically Informed Strategy to Enhance Pharmacist-Led Deprescribing in Care Homes for Older People
Source: Pharmacy (Basel). 2025 Sep 16;13(5):133. doi: 10.3390/pharmacy13050133 (PMC12452554; doi:10.3390/pharmacy13050133)
Supplement: Supplementary file 1 [file pharmacy-13-00133-s001.zip › pharmacy-3820173-supplementary/Supplementary file 1 Interview topic guide-1.pdf]

Version 4 12.04.21

CHIPPS Care Home Independent Pharmacist Prescribing Study

'Learning from CHIPPS – moving to policy'

Interview Topic Guide

Introduction

Thank you for agreeing to take part in the interview. We want to understand your perspectives on deprescribing in care homes. You may draw on your experiences of taking part in the CHIPPS service and any other deprescribing activity you have been part of.

I would like to highlight the confidentiality of everything you tell me, and specifically that:

- the recording will be deleted after being transcribed
- you won't be identified individually in any report.
- all information will be anonymised.
- we will not tell anyone else including your employer organisation, what you tell us as an individual.
- likewise no other individual e.g. GP, care home staff, resident will be identifiable in any report.
- all of your views are of value to us. There are no right or wrong answers,
- please ask me to clarify if the question isn't clear.
- we remind you not to share any personal or patient identifying information during this interview

Before we start I would like to clarify that by deprescribing we are talking about activity that leads to a medication being stopped, or reduced with the intention of alleviating potential harm. We are particularly interested in proactive deprescribing rather than reactive deprescribing where there has been clinical need

Anchor question	Potential probes	Links to TDF
**How does deprescribing fit in with your role?**<br><br>**What do you think about deprescribing being a part of your role?**	GP: opportunities for proactive deprescribing<br><br>CH staff: opportunities to consider proactive deprescribing<br><br>PIPs: opportunities for proactive deprescribing, competence	Professional identify<br><br>Beliefs about capabilities
**Who else do you work with when you deprescribe?**	Whose opinions seek: family, resident, care home staff, GP , other practice staff, secondary care experts, community pharmacist, others	Social/professional roles<br><br>Social influences
**Please tell me about your thoughts on proactive deprescribing in care homes?**	What are benefits of proactive deprescribing (resident, DBI, for pharmacist, GP, CH staff more global benefits<br><br>What might be disadvantages (risks, harms, missed opportunities for alternative activities)<br><br>Draw out any practice examples	Knowledge<br><br>Motivations and Goals<br><br>Optimism<br><br>Beliefs about consequences
**What are the things that help you to deprescribing in care homes?**	Any social influences (seen it work, having, trust in deprescribing professional)	Beliefs about capabilities and consequences

	Are there any environmental factors or resources which positively affect deprescribing (Prescence of pharmacist or GP, time, how often medicines routinely reviewed)<br><br>Are there any attitudinal factors which positively affect deprescribing (staff, family, resident)<br><br>Are there any governance or policy factors which positively affect deprescribing	Environmental context and resources<br><br>Social influences, professional roles, Optimism
**What are the things that could help you do MORE deprescribing in care homes (**Pick up follow ups depending on above responses)	Any social influences (seen it work, having, trust in deprescribing professional)<br><br>Are there any environmental factors or resources which positively affect deprescribing (Prescence of pharmacist or GP, time, how often medicines routinely reviewed)<br><br>Are there any attitudinal factors which positively affect	Beliefs about capabilities and consequences<br><br>Environmental context and resources<br><br>Social influences, professional roles, Optimism

	deprescribing (staff, family, resident)<br><br>Are there any governance or policy factors which positively affect deprescribing	
**Please tell me what are the challenges that stop you from deprescribing in care homes? (tailor depending on responses above)**	Any social influences (beliefs about only happen end of life, may increase behaviour which challenge)<br><br>Are there any environmental factors or resources which affect deprescribing (Prescence of pharmacist or GP, time, how often medicines routinely reviewed)<br><br>Are there any organisational governance or policy factors which affect deprescribing<br><br>Are there any wider governance or policy factors which affect deprescribing	Social Influences<br><br>Beliefs about consequences<br><br>Environmental context and resources<br><br>Professional roles

	Any different challenges for the team (PIP, GP CH depending on interviewee<br><br>Within the team, organisation and beyond	
