# Supplementary material for: Developing a Theoretically Informed Strategy to Enhance Pharmacist-Led Deprescribing in Care Homes for Older People
Source: Pharmacy (Basel). 2025 Sep 16;13(5):133. doi: 10.3390/pharmacy13050133 (PMC12452554; doi:10.3390/pharmacy13050133)
Supplement: Supplementary file 1 [file pharmacy-13-00133-s001.zip › pharmacy-3820173-supplementary/Supplementary file 2 _Phase 3 Survey (1).pdf]

Strategies to support pharmacist prescribers to deprescribe in care homes

Page 1

Expert Panel to Select Strategies to Support Pharmacist Prescribers to Deprescribe in Care Homes: An initial survey of your thoughts ahead of the workshop.

Thank you for agreeing to be part of our expert panel. Please complete this survey by **14th November** as it will form the basis of the work we will do together at the workshop on 16th November. The survey should take less than an hour and there is the option of saving your responses. Anonymised replies will be used for report writing. If you have not already done so, please also complete the consent form: https://forms.office.com/r/f3Kp3E0qtg.

If you have any questions about completing the survey, contact Jeanette Blacklock J.Blacklock@uea.ac.uk

Purpose of the Survey

Earlier this year we conducted interviews with pharmacist prescribers who have worked

in care homes, general practitioners, and care home staff. The purpose of the interviews was to find out the things that hindered (barriers) and helped (enablers) a pharmacist to deprescribe (stopping) medicines that have more risks than benefits for care home residents. They told us that there were three main barriers and three main enablers. We want your help to design a support package to address these barriers and enablers to support pharmacists effectively to deprescribe medicines for care home residents in collaboration with care home staff and general practitioners.

For each barrier and enabler, we found in the interviews, we have used health psychology and behaviour change theory to put together a list of possible strategies that might address it. In this survey, we want your initial views on these strategies so that we can remove the ones that aren't likely to work and keep the best ones. The strategies are quite general at this stage, but at the workshop we will discuss the best strategies and ask for your ideas on how to implement these in practice.

Although the support package we are designing is for pharmacists, we are also asking care home staff and general practitioners what they think because deprescribing will require the pharmacist to work with them.

Page 2: Instructions

We will show you each barrier and enabler to pharmacists' deprescribing in turn, along with the potential strategies to address them.

For each strategy, we will ask whether you think it is likely to be:

1. **Practical** to put in place
2. **Effective** in addressing the thing that helps or hinders deprescribing
3. **Acceptable** to you and others who would be involved e.g. other staff, residents and family
4. **Safe** and free of undesirable consequences
5. **Equitable** so not likely to increase disparities between residents or across settings e.g. different ethnicities and gender

We've provided space after each strategy for you to document your thoughts. For strategies that you think are a good idea, we'd like you to use this space to note any ideas about how to implement it in practice.

There are no right or wrong responses so please record your immediate reactions.

Saving your responses

It should take less than an hour to complete this survey and we recommend that you complete it in one sitting. If you are unable to complete it at one sitting and you need to stop, please note that **the survey is not saved automatically -** you must click **Finish later** at the bottom of the survey page. You can then either provide your email address to receive a 'finish later' URL from the online surveys provider, or you can bookmark the 'finish later' link to be able to resume the survey.

Page 3

Barrier 1: Pharmacists are worried that residents, and/or their families may not want to stop medication

Strategy 1: A way of showing the pharmacist other pharmacists who have had discussions with residents and relatives in order to successfully deprescribe.

	Agree	Disagree
Practical to put in place	☐	☐
Effective at addressing the thing that helps or hinders deprescribing	☐	☐
Acceptable to you and others who would be involved	☐	☐
Safe and free of undesirable consequences	☐	☐
Equitable so not likely to increase disparities between residents or across settings e.g., different ethnicities and gender	☐	☐

Please add any comments to support your decision or any ideas about how the strategy might work in practice.

Strategy 2: Tell pharmacists that the vast majority of residents and their family are open to having a medicine deprescribed by them.

	Agree	Disagree
Practical to put in place	☐	☐

Effective at addressing the thing that helps or hinders deprescribing	☐	☐
Acceptable to you and others who would be involved	☐	☐
Safe and free of undesirable consequences	☐	☐
Equitable so not likely to increase disparities between residents or across settings e.g., different ethnicities and gender	☐	☐

Please add any comments to support your decision or any ideas for how you'd like this strategy to look in practice

Strategy 3: Arrange for pharmacists to receive encouraging support from a colleague when engaging with residents and their families in deprescribing discussions.

	Agree	Disagree
Practical to put in place	☐	☐
Effective at addressing the thing that helps or hinders deprescribing	☐	☐
Acceptable to you and others who would be involved	☐	☐
Safe and free of undesirable consequences	☐	☐
Equitable so not likely to increase disparities between residents or across settings e.g., different ethnicities and gender	☐	☐

Please add any comments to support your decision, or any ideas about how the strategy might work in practice.

Strategy 4: Arrange for pharmacists to receive practical help from a colleague when engaging with residents and their families in deprescribing discussions.

	Agree	Disagree
Practical to put in place	☐	☐
Effective at addressing the thing that helps or hinders deprescribing	☐	☐
Acceptable to you and others who would be involved	☐	☐
Safe and free of undesirable consequences	☐	☐
Equitable so not likely to increase disparities between residents or across settings e.g., different ethnicities and gender	☐	☐

Please add any comments to support your decision, or any ideas about how the strategy might work in practice.

Strategy 5: Arrange praise for pharmacists who are successfully engaging residents and their families in deprescribing discussions.

	Agree	Disagree
Practical to put in place	☐	☐

Effective at addressing the thing that helps or hinders deprescribing	☐	☐
Acceptable to you and others who would be involved	☐	☐
Safe and free of undesirable consequences	☐	☐
Equitable so not likely to increase disparities between residents or across settings e.g., different ethnicities and gender	☐	☐

Please add any comments to support your decision, or any ideas about how the strategy might work in practice.

Page 4

Barrier 2: Pharmacists think that deprescribing is generally riskier than continuing to prescribe a medication, even if there are no anticipated future gains.

Strategy 1: A way of showing pharmacists that deprescribing inappropriate medication leads to professional satisfaction e.g. seeing a resident clinically improving.

	Agree	Disagree
Practical to put in place	☐	☐
Effective at addressing the thing that helps or hinders deprescribing	☐	☐
Acceptable to you and others who would be involved	☐	☐
Safe and free of undesirable consequences	☐	☐
Equitable so not likely to increase disparities between residents or across settings e.g., different ethnicities and gender	☐	☐

Please add any comments to support your decision, or any ideas about how the strategy might work in practice.

Strategy 2: Emphasise the benefits of deprescribing and harmful consequences of failing to deprescribe in terms which will resonate with pharmacists.

	Agree	Disagree

Practical to put in place	☐	☐
Effective at addressing the thing that helps or hinders deprescribing	☐	☐
Acceptable to you and others who would be involved	☐	☐
Safe and free of undesirable consequences	☐	☐
Equitable so not likely to increase disparities between residents or across settings e.g., different ethnicities and gender	☐	☐

Please add any comments to support your decision, or any ideas about how the strategy might work in practice.

Strategy 3: A way of showing the pharmacist about the positive health consequences for the patient of deprescribing inappropriate medication and/or the negative health consequences of failing to deprescribe.

	Agree	Disagree
Practical to put in place	☐	☐
Effective at addressing the thing that helps or hinders deprescribing	☐	☐
Acceptable to you and others who would be involved	☐	☐
Safe and free of undesirable consequences	☐	☐
Equitable so not likely to increase disparities between residents or across settings e.g., different ethnicities and gender	☐	☐

Please add any comments to support your decision, or any ideas about how the strategy might work in practice.

Strategy 4: A way of showing pharmacists the wider positive outcomes of deprescribing inappropriate medication and/or the wider negative outcomes of failing to deprescribe e.g. financial savings for the NHS.

	Agree	Disagree
Practical to put in place	☐	☐
Effective at addressing the thing that helps or hinders deprescribing	☐	☐
Acceptable to you and others who would be involved	☐	☐
Safe and free of undesirable consequences	☐	☐
Equitable so not likely to increase disparities between residents or across settings e.g., different ethnicities and gender	☐	☐

Please add any comments to support your decision, or any ideas about how the strategy might work in practice.

Strategy 5: Advise pharmacists to list and compare the advantages and disadvantages of deprescribing inappropriate medication for care home residents.

Please don't select more than 1 answer(s) per row.

Please select at least 5 answer(s).

	Agree	Disagree
Practical to put in place	☐	☐
Effective at addressing the thing that helps or hinders deprescribing	☐	☐
Acceptable to you and others who would be involved	☐	☐
Safe and free of undesirable consequences	☐	☐
Equitable so not likely to increase disparities between residents or across settings e.g., different ethnicities and gender	☐	☐

Please add any comments to support your decision, or any ideas about how the strategy might work in practice.

Page 5

Barrier 3: Pharmacists are worried that some care home staff may be resistant to deprescribing

Strategy 1: A way of showing the pharmacist that other pharmacists are successfully working with care home staff.

	Agree	Disagree
Practical to put in place	☐	☐
Effective at addressing the thing that helps or hinders deprescribing	☐	☐
Acceptable to you and others who would be involved	☐	☐
Safe and free of undesirable consequences	☐	☐
Equitable so not likely to increase disparities between residents or across settings e.g., different ethnicities and gender	☐	☐

Please add any comments to support your decision, or any ideas about how the strategy might work in practice.

Strategy 2: Provide evidence to the pharmacist that the vast majority of care home staff are supportive of deprescribing.

	Agree	Disagree
Practical to put in place	☐	☐

Effective at addressing the thing that helps or hinders deprescribing	☐	☐
Acceptable to you and others who would be involved	☐	☐
Safe and free of undesirable consequences	☐	☐
Equitable so not likely to increase disparities between residents or across settings e.g., different ethnicities and gender	☐	☐

Please add any comments to support your decision, or any ideas about how the strategy might work in practice.

Strategy 3: Arrange for pharmacists to receive encouragement from a colleague to work with care home staff to deprescribe.

	Agree	Disagree
Practical to put in place	☐	☐
Effective at addressing the thing that helps or hinders deprescribing	☐	☐
Acceptable to you and others who would be involved	☐	☐
Safe and free of undesirable consequences	☐	☐
Equitable so not likely to increase disparities between residents or across settings e.g., different ethnicities and gender	☐	☐

Please add any comments to support your decision, or any ideas about how the strategy might work in practice.

Strategy 4: Arrange for pharmacists to receive practical help from a colleague to work with care home staff to deprescribe.

	Agree	Disagree
Practical to put in place	☐	☐
Effective at addressing the thing that helps or hinders deprescribing	☐	☐
Acceptable to you and others who would be involved	☐	☐
Safe and free of undesirable consequences	☐	☐
Equitable so not likely to increase disparities between residents or across settings e.g., different ethnicities and gender	☐	☐

Please add any comments to support your decision, or any ideas about how the strategy might work in practice.

Strategy 5: Arrange praise for pharmacists who are successfully engaging with care home staff in deprescribing discussions.

	Agree	Disagree
Practical to put in place	☐	☐

Effective at addressing the thing that helps or hinders deprescribing	☐	☐
Acceptable to you and others who would be involved	☐	☐
Safe and free of undesirable consequences	☐	☐
Equitable so not likely to increase disparities between residents or across settings e.g., different ethnicities and gender	☐	☐

Please add any comments to support your decision, or any ideas about how the strategy might work in practice.

Page 6

Enabler 1: Pharmacists believe that deprescribing for residents will lead to benefits

Strategy 1: Reward pharmacists who are successfully deprescribing.

	Agree	Disagree
Practical to put in place	☐	☐
Effective at addressing the thing that helps or hinders deprescribing	☐	☐
Acceptable to you and others who would be involved	☐	☐
Safe and free of undesirable consequences	☐	☐
Equitable so not likely to increase disparities between residents or across settings e.g., different ethnicities and gender	☐	☐

Please add any comments to support your decision, or any ideas about how the strategy might work in practice.

Strategy 2: Arrange praise for pharmacists whose deprescribing positively impacts a resident's health and/or wellbeing.

	Agree	Disagree
Practical to put in place	☐	☐
Effective at addressing the thing that helps or hinders deprescribing	☐	☐

	Agree	Disagree
Acceptable to you and others who would be involved	☐	☐
Safe and free of undesirable consequences	☐	☐
Equitable so not likely to increase disparities between residents or across settings e.g., different ethnicities and gender	☐	☐

Please add any comments to support your decision, or any ideas about how the strategy might work in practice.

Strategy 3: Tell pharmacists that they will get a reward if they successfully deprescribe.

	Agree	Disagree
Practical to put in place	☐	☐
Effective at addressing the thing that helps or hinders deprescribing	☐	☐
Acceptable to you and others who would be involved	☐	☐
Safe and free of undesirable consequences	☐	☐
Equitable so not likely to increase disparities between residents or across settings e.g., different ethnicities and gender	☐	☐

Please add any comments to support your decision, or any ideas about how the strategy might work in practice.

Page 7

Enabler 2: Pharmacists see deprescribing as a key part of their professional role

Strategy 1: Arrange for pharmacists to receive encouragement to deprescribe.

	Agree	Disagree
Practical to put in place	☐	☐
Effective at addressing the thing that helps or hinders deprescribing	☐	☐
Acceptable to you and others who would be involved	☐	☐
Safe and free of undesirable consequences	☐	☐
Equitable so not likely to increase disparities between residents or across settings e.g., different ethnicities and gender	☐	☐

Please add any comments to support your decision, or any ideas about how the strategy might work in practice.

Strategy 2: Draw attention to pharmacists who are deprescribing and see it as part of their role to encourage others to think the same.

	Agree	Disagree
Practical to put in place	☐	☐
Effective at addressing the thing that helps or hinders deprescribing	☐	☐

Acceptable to you and others who would be involved	☐	☐
Safe and free of undesirable consequences	☐	☐
Equitable so not likely to increase disparities between residents or across settings e.g., different ethnicities and gender	☐	☐

Please add any comments to support your decision, or any ideas about how the strategy might work in practice.

Strategy 3: Have an influential figure endorse that deprescribing is a part of a pharmacist's role e.g. president of a professional body.

	Agree	Disagree
Practical to put in place	☐	☐
Effective at addressing the thing that helps or hinders deprescribing	☐	☐
Acceptable to you and others who would be involved	☐	☐
Safe and free of undesirable consequences	☐	☐
Equitable so not likely to increase disparities between residents or across settings e.g., different ethnicities and gender	☐	☐

Please add any comments to support your decision, or any ideas about how the strategy might work in practice.

Strategy 4: Encourage pharmacists to identify that that they are ‘deprescribers’, in a similar fashion to ‘flu fighters’ or ‘antibiotic guardians’.

	Agree	Disagree
Practical to put in place	☐	☐
Effective at addressing the thing that helps or hinders deprescribing	☐	☐
Acceptable to you and others who would be involved	☐	☐
Safe and free of undesirable consequences	☐	☐
Equitable so not likely to increase disparities between residents or across settings e.g., different ethnicities and gender	☐	☐

Please add any comments to support your decision, or any ideas about how the strategy might work in practice.

Page 8

Enabler 3: Endorsement by the general practitioner supports pharmacist deprescribing

Strategy 1: General practitioners provide encouragement to pharmacists to deprescribe.

	Agree	Disagree
Practical to put in place	☐	☐
Effective at addressing the thing that helps or hinders deprescribing	☐	☐
Acceptable to you and others who would be involved	☐	☐
Safe and free of undesirable consequences	☐	☐
Equitable so not likely to increase disparities between residents or across settings e.g., different ethnicities and gender	☐	☐

Please add any comments to support your decision, or any ideas about how the strategy might work in practice.

Strategy 2: General practitioners practically support pharmacists to deprescribe e.g. flag care home residents who might benefit from deprescribing.

	Agree	Disagree
Practical to put in place	☐	☐

Effective at addressing the thing that helps or hinders deprescribing	☐	☐
Acceptable to you and others who would be involved	☐	☐
Safe and free of undesirable consequences	☐	☐
Equitable so not likely to increase disparities between residents or across settings e.g., different ethnicities and gender	☐	☐

Please add any comments to support your decision, or any ideas about how the strategy might work in practice.

Strategy 3: A way of showing pharmacists that other pharmacists receive general practitioner endorsement.

	Agree	Disagree
Practical to put in place	☐	☐
Effective at addressing the thing that helps or hinders deprescribing	☐	☐
Acceptable to you and others who would be involved	☐	☐
Safe and free of undesirable consequences	☐	☐
Equitable so not likely to increase disparities between residents or across settings e.g., different ethnicities and gender	☐	☐

Please add any comments to support your decision, or any ideas about how the strategy might work in practice.

Strategy 4: A way of showing pharmacists that general practitioners approve of deprescribing being a part of their role.

	Agree	Disagree
Practical to put in place	☐	☐
Effective at addressing the thing that helps or hinders deprescribing	☐	☐
Acceptable to you and others who would be involved	☐	☐
Safe and free of undesirable consequences	☐	☐
Equitable so not likely to increase disparities between residents or across settings e.g., different ethnicities and gender	☐	☐

Please add any comments to support your decision, or any ideas about how the strategy might work in practice.

Strategy 5: Arrange for general practitioners to praise pharmacists who are successfully deprescribing.

	Agree	Disagree
Practical to put in place	☐	☐

Effective at addressing the thing that helps or hinders deprescribing	☐	☐
Acceptable to you and others who would be involved	☐	☐
Safe and free of undesirable consequences	☐	☐
Equitable so not likely to increase disparities between residents or across settings e.g., different ethnicities and gender	☐	☐

Please add any comments to support your decision, or any ideas about how the strategy might work in practice.

Page 9: You're all finished!

Thank you for completing this survey.

We look forward to seeing you at the workshop on Tuesday 16 November at 9.30 am.
