# Supplementary material for: Developing a Theoretically Informed Strategy to Enhance Pharmacist-Led Deprescribing in Care Homes for Older People
Source: Pharmacy (Basel). 2025 Sep 16;13(5):133. doi: 10.3390/pharmacy13050133 (PMC12452554; doi:10.3390/pharmacy13050133)
Supplement: Supplementary file 1 [file pharmacy-13-00133-s001.zip › pharmacy-3820173-supplementary/Supplementary file 3 Mapping interview data to the Theoretical Domains Framework-1.pdf]

Example of Mapping interview data to the Theoretical Domains Framework

**Thematic analysis:**<br><br>**Theme**	**Mapping interview data to TDF:**<br><br>**Behaviour Belief**	**A barrier behaviour**	**Interview quote**	**An enabler behaviour**	**Interview quote**
Personal competency	Pharmacists should only deprescribe in their clinical speciality	Nervous about deprescribing some types of medicines for some types of clinical conditions	*'I am a cardiac pharmacist, if it was a pain med I would discuss with the GP, if it was respiratory, I would discuss with respiratory nurse' PIP_9*	Pharmacists have the clinical knowledge to deprescribe	*'…knowledge about what we can reduce, what we can just stop and it's where my mind goes' PIP_1*
Personal competency	Pharmacists need extra training to deprescribe	Pharmacists may not have the training to deprescribe	*'More training is needed, there's only so much on a guideline, and it needs more you can't list every single thing, about why you wouldn't want to deprescribe something'<br>PIP_16*	Confidence grows after training	*'geriatrician come and gave a talk about things and I'm aware of the types you can look up risks and benefits but I don't tend to use that in routine practice now' PIP_21*
Knowing resident	Pharmacists need to understand the clinical context of the medicine and the patient	Not having access to clinical notes	*'Be cautious not make decisions without background knowledge'<br>PIP_23*	Need to know what medicine is indicated for	*'Looking at the person, what's going on with the patient, what's their prognosis, what's it indicated for, what risks would be to if we removed that'<br><br>PIP_10*

Frailty of resident	Medicines act differently in frail, older people	General pharmaceutical guidance not tailored to older people'	*'There is guidance but it only goes up to a certain age..*<br>PIP_14	Knowledge that medicines affect frail, older people differently	*'Low blood pressure in the over 90s is actually a risk factor for them, and causes more deaths than having slightly high blood pressure' PIP_20*
