# Supplementary material for: Developing a Theoretically Informed Strategy to Enhance Pharmacist-Led Deprescribing in Care Homes for Older People
Source: Pharmacy (Basel). 2025 Sep 16;13(5):133. doi: 10.3390/pharmacy13050133 (PMC12452554; doi:10.3390/pharmacy13050133)
Supplement: Supplementary file 1 [file pharmacy-13-00133-s001.zip › pharmacy-3820173-supplementary/supplementary file 4 example consensus workshop discussion.pdf]

Group 2

Activity 1
30 min.

Small group discussion:

Designing the strategy:

Emphasise the benefits of deprescribing and harmful consequences of failing to deprescribe in terms which will resonate with pharmacists.

In this first activity, we will be discussing the barrier and strategy:

Pharmacists think that deprescribing is generally riskier than continuing to prescribe a medication, even if there are no anticipated future gains.

→

Emphasise the benefits of deprescribing and harmful consequences of failing to deprescribe in terms which will resonate with pharmacists.

Activity instructions

- Key things to think about:
  - What could the strategy look like when implemented (content)?
  - How might the strategy be delivered?
  - How often and/or for how long?

Nominate a spokesperson to feed back

Activity 1 – Group 2 feedback notes page 1

Pharmacists think that deprescribing is generally riskier than continuing to prescribe a medication, even if there are no anticipated future gains.

→

Emphasise the benefits of deprescribing and harmful consequences of failing to deprescribe in terms which will resonate with pharmacists.

Notes

- Case studies – real life – anonymized to enable pharmacist to relate to them – not fake
    - Help pharmacist consider all other aspects e.g. social prescribing
    - Demedicalise the activity
- Statistics in number with respect to negative outcomes
- Stories / Patient narratives +ve and –ve / stories
- Examples of where things went wrong
- Reflect on own case studies on what happened over time
- Showcasing partnership approach and support each partner can give each other – Not just down to me
- Reduction of antipsychotics and introduction of more psychosocial interventions which care team could implement
- Increasing frailty changes risk benefit

Activity 1 – Group 2

Pharmacists think that deprescribing is generally riskier than continuing to prescribe a medication, even if there are no anticipated future gains.

→

Emphasise the benefits of deprescribing and harmful consequences of failing to deprescribe in terms which will resonate with pharmacists.

Notes

- How communicated – what platform
- IT systems and include as training via platforms and providing prompts
- Prepare pharmacists for role – awareness of social prescribing and other local initiatives
- Initiatives within the care home provider – wellbeing and dementia care strategies e.g. music therapy
- PCN pharmacist training program – embed within that for those pharmacists
- CPPE training packages – deprescribing module review – about safe use of medication – not just for pharmacists in care homes
- Local and national approach
- Supportive local network – peer to peer support – space for reflective learning together
- Easy to put on-line and click through them – get saturated with these – something more meaningful preferred – small groups of work with genuine reflection – share experiences/stories – take own reflective case studies to the session
- Teams to set up own network – forums using IT systems
- Challenge is useful – make people to consider all elements
- Training with care home staff interested in medicines preferable to training in professional isolation
- Care staff do often lead on deprescribing discussions

Activity 1 – Group 2 Feedback notes page 2

Pharmacists think that deprescribing is generally riskier than continuing to prescribe a medication, even if there are no anticipated future gains.

→

Emphasise the benefits of deprescribing and harmful consequences of failing to deprescribe in terms which will resonate with pharmacists.

Notes

- Use a dashboard with update on performance
    - Number of medicines stopped
    - Number of falls
- Reinforce practice and may reduce need for more training
- Set up community of practice
    - Continual learning and development
    - Quarterly
        - New fresh case studies
        - Building evidence base picked up here
        - Make deprescribing one of the elements
    - Network where people can post experiences/evidence/ideas/updates

Pre-workshop activity
Results

Showing real life evidence of failing to deprescribe e.g. case based discussions, data from hospital admissions, significant events

Communicate risks and benefits for high risk medicines with diagrams red/green faces. Could then use these in the consultations

Patient/family/care home staff feedback after deprescribing discussions e.g. video interviews or written responses

Group 2

Activity 2

30 min.

Small group discussion:

Designing the strategy:

Arrange for pharmacists to receive encouragement to deprescribe.

In this second activity, we will be discussing the enabler and strategy:

Pharmacists see deprescribing as a key part of their professional role. → **Arrange for pharmacists to receive encouragement to deprescribe.**

Activity instructions

- Key things to think about:
  - What could the strategy look like when implemented (content)?
  - How might the strategy be delivered?
  - How often and/or for how long?

Nominate a spokesperson to feed back

Activity 2 – Group 2 Feedback notes Page 1

Pharmacists see deprescribing as a key part of their professional role.

→

Arrange for pharmacists to receive encouragement to deprescribe.

Notes

- Sharing good practice
- Not to highlight amount of deprescribing – want the right activity not the most activity
- Sharing successful practice
- Knowing the impact of deprescribing by celebrating impact on patient
- Work with a mentor/buddy who works in another care home
    - Role model
- Updating pharmacists about outcome down the line so they can see whether there was success
- Community of practice idea works here
- Dashboard giving automated feedback
    - Keep it simple not cumbersome – use existing data
    - Care home based – also include good practices such as hydration levels – not monetary orientated
    - Recorded in electronic care plans anyway so shouldn't be too difficult to enable immediate feedback
    - Pharmacist needs to know about this (back to point about follow on result)
- Pharmacists need to know home, staff, residents, relatives

Activity 2 – Group 2 Feedback notes page 2

Pharmacists see deprescribing as a key part of their professional role. → **Arrange for pharmacists to receive encouragement to deprescribe.**

Strategy

- Role of relatives in the process of encouraging pharmacists to deprescribe
- Relatives always forthcoming and want to be engaged in activities – welcome opportunity
    - Address barrier of relative reaction to deprescribing
- Care home team conduit to involving relatives
- Provide pharmacists with access to evidence-based tools to support safe deprescribing e.g. STOPP. START
- Talk about Bannerjee report regarding overuse of psychotic treatments
- Legislation to be considered – how managed internationally e.g. antipsychotics
- Consider role of regulator and how they can encourage the right practice
- Quality improvement projects to encourage the right behaviours

How often or how long

- Ongoing feedback (Dashboard)
- Getting software to work for the user not the user to work for the software
- Community of practice
- Frequency of review to be considered but quarterly better than annual and feedback in time

Pre-workshop activity
Results

Circulating positive deprescribing cases where pharmacists have made a big impact on improving care

Targeted quality improvement projects promoting deprescribing of specific potentially harmful medications that are commonly over prescribed
