# Supplementary material for: Developing a Theoretically Informed Strategy to Enhance Pharmacist-Led Deprescribing in Care Homes for Older People
Source: Pharmacy (Basel). 2025 Sep 16;13(5):133. doi: 10.3390/pharmacy13050133 (PMC12452554; doi:10.3390/pharmacy13050133)
Supplement: Supplementary file 1 [file pharmacy-13-00133-s001.zip › pharmacy-3820173-supplementary/supplementary file 5 policy brief.pdf]

Policy brief

Embedding pharmacist deprescribing into care homes to improve resident medicines safety

Why is this important?

- 50% of care home residents are prescribed a medicine that is unnecessary or harmful
- Deprescribing unnecessary or harmful medicines reduces hospitalisation and deaths
- Failure to embed deprescribing in care homes contributes to overprescribing

Relevance to policy

World Health Organization

Global Patient Safety Challenge: Medication Without Harm

Reduce harm from medicines by 50% and create a safer care home setting

Department of Health & Social Care

National Overprescribing review

A plan to reduce overprescribing to make patient care better and safer

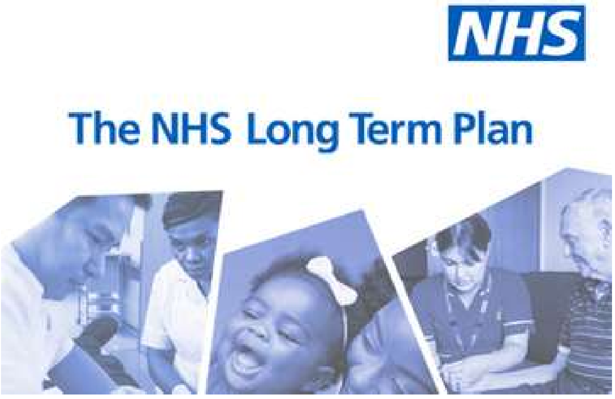

Employ 8400 clinical pharmacists in primary care

Key messages for policy

To support Clinical Pharmacists to deprescribe in care homes:

Greater emphasis on deprescribing within pharmacists' education, training and role

Direct employment in General Practices will increase pharmacists' effectiveness to deprescribe harmful medicines in care homes

QUEEN'S UNIVERSITY BELFAST
UNIVERSITY OF ABERDEEN
UNIVERSITY OF LEICESTER
UNIVERSITY OF LEEDS

Key messages for practice

To support Clinical Pharmacists to deprescribe in care homes:

Deprescribing demostrations

Of succesful pharmacist interactions with care home residents, famly and staff

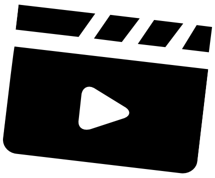

Mentorship for pharmacists

From an experienced colleague

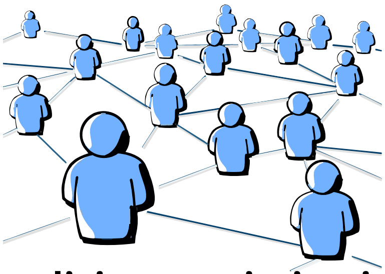

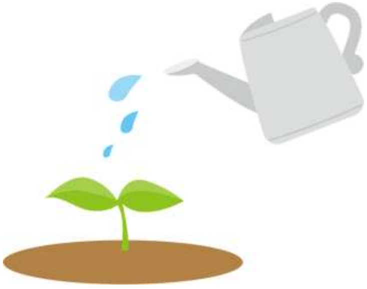

Establish medicines optimisation networks

Focussing on care homes to share best practice

What is the evidence for these messages?

The Care Homes Independent Pharmacist Prescribing Study (CHIPPS) (2015-2020) found that Prescribing Pharmacists who had completed an evidence-based training programme were effective at deprescribing unnecessary or potentially harmful medicines for care home residents.

- **Deprescribing focussed on risk medicinessuch as antipsychotics and benzodiazepines**
- **Pharmacists employment by a medical practice deprescribed on average over 5 more medicines per resident than those who were employed by other organisations.**

The things that help and hinder Pharmacists Prescribers to deprescribe are:

Thing's that help:

Recognising deprescribing as part of a pharmacist's role

Being shown the benefits of deprescribing and

Being supported by a mentor

Things that hinder:

Not having enough time for deprescribing

Thinking that people won't approve of deprescribing

Concerns about the consequences of deprescribing

For more information contact

Dr Sion Scott
University of Leicester
s.scott@le.ac.uk

Professor David Wright
University of Leicester
d.j.wright@le.ac.uk

FUNDED BY
NIHR | National Institute for Health Research

NHS
Norfolk and Waveney
Clinical Commissioning Group

© 2022, University of Leicester. All rights reserved.
